# The Determinants of HIV Treatment Costs in Resource Limited Settings

**DOI:** 10.1371/journal.pone.0048726

**Published:** 2012-11-07

**Authors:** Nicolas A. Menzies, Andres A. Berruti, John M. Blandford

**Affiliations:** 1 U.S. Centers for Disease Control and Prevention, Atlanta, Georgia, United States of America; 2 ICF-Macro Inc, Atlanta, Georgia, United States of America; 3 Center for Health Decision Science, Harvard School of Public Health, Boston, Massachusetts, United States of America; Boston University, United States of America

## Abstract

**Background:**

Governments and international donors have partnered to provide free HIV treatment to over 6 million individuals in low and middle-income countries. Understanding the determinants of HIV treatment costs will help improve efficiency and provide greater certainty about future resource needs.

**Methods and Findings:**

We collected data on HIV treatment costs from 54 clinical sites in Botswana, Ethiopia, Mozambique, Nigeria, Uganda, and Vietnam. Sites provided free HIV treatment funded by the U.S. President’s Emergency Plan for AIDS Relief (PEPFAR), national governments, and other partners. Service delivery costs were categorized into successive six-month periods from the date when each site began HIV treatment scale-up. A generalized linear mixed model was used to investigate relationships between site characteristics and per-patient costs, excluding ARV expenses. With predictors at their mean values, average annual per-patient costs were $177 (95% CI: 127–235) for pre-ART patients, $353 (255–468) for adult patients in the first 6 months of ART, and $222 (161–296) for adult patients on ART for >6 months (excludes ARV costs). Patient volume (no. patients receiving treatment) and site maturity (months since clinic began providing treatment services) were both strong independent predictors of per-patient costs. Controlling for other factors, costs declined by 43% (18–63) as patient volume increased from 500 to 5,000 patients, and by 28% (6–47) from 5,000 to 10,000 patients. For site maturity, costs dropped 41% (28–52) between months 0–12 and 25% (15–35) between months 12–24. Price levels (proxied by per-capita GDP) were also influential, with costs increasing by 22% (4–41) for each doubling in per-capita GDP. Additionally, the frequency of clinical follow-up, frequency of laboratory monitoring, and clinician-patient ratio were significant independent predictors of per-patient costs.

**Conclusions:**

Substantial reductions in per-patient service delivery costs occur as sites mature and patient cohorts increase in size. Other predictors suggest possible strategies to reduce per-patient costs.

## Introduction

In 2009, there was $US15·9 billion spent globally on HIV control, the majority in developing countries [1]. Most of this funding was devoted to providing care and antiretroviral therapy for infected individuals, and the expansion of treatment services has been one of the successes of global HIV control. The emphasis on treatment within HIV control programs will likely grow as programs adopt more aggressive antiretroviral therapy (ART) initiation criteria [2] and as evidence accumulates about the substantial prevention benefits resulting from HIV treatment [3]. Despite these advances, treatment programs face constrained budgets, with both domestic and external HIV spending under increasing pressure.

Obtaining better evidence on HIV treatment costs is a priority of major donors such as the President’s Emergency Plan for AIDS Relief (PEPFAR), the Global Fund and the Gates Foundation [4], [5]. Understanding the costs of HIV treatment serves two important functions. The first is to plan for future expenditure requirements: as HIV treatment requires lifelong care, initiation of patients on treatment implies a resource commitment both in the present and future. Gaining greater certainty about resource requirements puts funders in a better position to make long-term commitments about program targets. The second function is to suggest strategies for improving the efficiency of HIV treatment programs. Progressive gains have been made in driving down the costs of antiretroviral drugs, with a first-line regimen that cost $1,200 in 2001 now available for $120 [6], [7]. While antiretroviral drug (ARV) costs historically consumed the majority of a treatment program’s budget this is no longer the case, with non-ARV costs–personnel, facilities, laboratory tests–consuming a progressively larger proportion of the treatment budget as the annual cost of an ARV regimen has dropped. Understanding these service delivery costs is important as treatment programs seek further efficiencies.

Evidence on patient-level determinants of treatment costs has been accumulating, with disease severity [8], drug regimen [9], route of infection and sociodemographic characteristics [10] all shown to produce differences in annual treatment costs. Less evidence is available on programmatic determinants of treatment costs, despite large variation in average cost observed between providers [11], [12]. Part of the reason for this evidence gap is heterogeneity in costing methods between studies: as most studies only include one or a small number of sites, data must be pooled across studies to gain sufficient power to investigate site-level variables, yet variation in costing approach between studies hampers such pooled analyses. An increasing number of modeled analyses have provided insight into the consequences of policies such as ART initiation criteria [13]–[15], first-line drug regimens [16], and laboratory tests [14], [17], yet by their design these analyses only address cost differentials resulting from the frequency with which individual services are provided (e.g., inpatient days, CD4 count tests), and cannot provide information on the factors determining the unit costs of these services. Some modeled analyses have attempted to describe the cost functions of scaling up HIV treatment programs, but are hampered by the relative lack of empirical evidence to parameterize these functions [18], [19]. A small number of empirical studies in South Africa have investigated site-level cost determinants, finding input prices [20], patient volume [20], site maturity [21], staffing approach [22], and location [23] all to influence average treatment costs, yet these findings are necessarily tentative given the small sample of sites–between one and four–included in these studies.

This study makes use of a unique dataset on HIV treatment costs drawn from 54 HIV treatment sites across six countries, collected using consistent methods and covering a comprehensive range of HIV treatment services [12], [24]. The common methodology allows valid comparisons across sites, and across time periods within each site. We used these data to investigate site-level determinants of the service delivery costs of HIV treatment.

## Methods

This analysis utilizes a dataset of HIV treatment costs collected from 54 HIV treatment sites across six countries. These data were collected as part of a multicountry costing study conducted in Botswana, Ethiopia, Nigeria, Uganda and Vietnam (9 sites per country), as well as a more recent study conducted in Mozambique (11 sites) that utilized the same costing methods. Two Ugandan sites were excluded from the analysis due to a lack of adequate patient volume data. All sites included in this dataset were out-patient clinics providing free treatment for HIV-infected individuals, and all relied on resource support from a mix of funders, including PEPFAR. Most sites were attached to a larger health facility in some way, with only 8 out of 54 sites being stand-alone clinics. Data collection adopted a comprehensive provider perspective, including the costs of personnel, drugs and other clinical supplies, laboratory supplies, other supplies, travel, utilities and building costs, training and supervision, equipment and renovation/construction. This perspective included the costs of any regular technical assistance supervision, M&E, and management support to the site, but excluded higher-level overhead costs incurred at a regional or central-level which could not be attributed directly to the site. Data were collected with a modified macro-costing approach, whereby total site-level costs were estimated for each patient type. These totals were divided by patient volume (no. patient-years) for each patient type to estimate the average cost per patient. For each site, data were collected retrospectively to cover the full duration of site activities from the time when sites began scaling up to provide HIV treatment to the time of data collection. Standardized data collection tools were used to extract information from accounting records, prescribing logs, equipment inventories, and routine reports, as well through structured interviews with site personnel. Individual cost items were coded to allow disaggregation by program activity, budget category and funder, and costs were broken into successive 6-month periods for analysis. Cost analyses included all patients receiving HIV treatment at study sites, including both ART and pre-ART patients, with data on patient volume drawn from routine program reporting records. By the end of the evaluation, a total of 76,416 ART patients and 95,538 pre-ART patients were receiving HIV care at study sites. The studies which collected these data received institutional review board clearance and data collection was conducted with the approval of the Ministry of Health in each country. Further detail on data collection methods is reported in Menzies, et al [12].

The subject of the analysis is the annualized per-patient cost of service delivery, excluding ARV expenditures. The costs of ARVs were excluded from the analysis as these can be better explained by global drug commodity trends, regimen distributions and national price levels, and are not principally driven by site-level factors. Following standard practice, primary data on resource use were converted to economic costs, with investments annualized over their useful life at a 3% discount rate, and donated items valued at market prices [25]. Overheads and shared costs were allocated by direct allocation [26] and the opportunity costs of existing infrastructure were estimated as the equivalent rental cost. All costs were converted to U.S. dollars using prevailing inter-bank exchange rates and inflated to current prices. Results are reported in 2010 U.S. dollars.

The duration for which data were available varied between sites, from 6 to 36 months, providing between 1 and 6 six-month periods for analysis (mean  = 3·0 periods per site). In addition, cost data were available for five distinct patient types: pre-ART patients, newly initiated adult ART patients, established adult ART patients, newly initiated pediatric ART patients, and established pediatric ART patients. Pediatric and adult patients were those aged 0–15 and >15 years respectively, and newly initiated and established patients were those who had received ART for 0–6 and >6 months, respectively.

Possible explanatory variables were divided into distal determinants and proximal determinants. Distal determinants described general features of the site (i.e., location, health system level, type of administration) that might play a role in determining the operating characteristics of the site and thus influence costs. Proximal variables described site operating characteristics (i.e., site maturity, patient volume, frequency of clinical and laboratory monitoring, comprehensiveness of care services provided, staffing structure, percentage of spending devoted to management and administration, and log per-capita GDP as an indicator of price levels). The first part of the analysis focuses on proximal determinants, the second part focuses on distal determinants. Table 1 provides descriptions for explanatory variables included in the analysis.

**Table 1:** Variable descriptions.

Variable Name	Description
***Proximal Determinants***	
Site Maturity	Number of months since sites began scaling-up to provide HIV treatment, calculated from the midpoint of each costing period.
Patient Volume	Average number of patients (combined ART + pre-ART) supported by the site during the costing period, in 1,000 s.
Clinic Visit Frequency	Average number of clinic visits (per patient) during 6-month period (differs by patient type).
CD4 Count Frequency	Average number of CD4 count tests (per patient) during 6-month period (differs by patient type).
Comprehensiveness of Care	Index of the comprehensiveness of patient care, the sum of the number of additional care services provided at the treatment site, from the following list of priority services: onsite TB treatment, isoniazid preventive therapy for TB, STI treatment, cotrimoxazole prophylaxis, provision of insecticide treated bednets, provision of water sanitation products, psychosocial support, pain management, end of life care, availability of viral load testing, and community follow-up of patients missing appointments. In our sample the index varied from 2 to 10 with a mean of 6·4.
Clinician:Patient Ratio	Number of full-time equivalent (FTE) clinicians per 1,000 patients. Clinician includes physicians, nurses, clinical officers, medical assistants, clinical counselors, and other non-physician clinicians.
Doctor:Clinician Ratio	FTE physicians as a percentage of all clinicians, included to investigate task-shifting issues.
Pct Mgmt/Admin	Percent of total resources devoted to site-level management and administration activities (c.f. direct patient care).
Log Per Capita GDP	Natural log of annual per-capita GDP in U.S. dollars [27], as an indicator of local price levels and complexity of resource use [28].
Patient Type	Indicator variables for patient type, including pre-ART, established adult ART (reference category), newly initiated adult ART, established pediatric ART, and newly initiated pediatric ART.
***Distal Determinants***	
Location	Indicator variables for site location, dichotomized into urban and non-urban (reference category). The sample included 39 urban sites and 15 non-urban sites.
Health System Level	Indicator variables for health system level, including primary (reference category), secondary, and tertiary sites. The sample included 10 primary sites, 16 secondary sites and 28 tertiary sites.
Type of Administration	Indicator variables for type of administration, dichotomized into government and NGO/FBO (reference category). The sample included 46 government sites and 8 NGO/FBO sites.

The dataset has a complex structure and a generalized linear mixed model (GLMM) was adopted for the analysis, with a log link function, and random effect terms used to account for clustering at country, site, and time period level. Fixed effects were also included for each patient type. The dataset includes a total of 692 observations, however the effective sample size is smaller than this suggests due to the clustering at site and time period level. The model was estimated using Markov Chain Monte Carlo (MCMC) simulation implemented with **R** statistical computing software [29], [30].

The estimates produced by the GLMM regression relate to log-transformed costs, and care must be taken when interpreting coefficient values. Individual regression coefficients have a non-linear relationship with the raw per-patient cost, such that a unit increase in a particular predictor x_i_ (with regression coefficient β_i_) results in an average per-patient cost that is 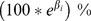 of its original value, all other values being held equal. For this reason a series of first differences was calculated to investigate the implications of changes in site characteristics for the average per-patient cost, by simulating the absolute and percentage change in per-patient costs resulting from the change in one explanatory variable, all other variables being held at their mean value.

Direct retransformation of logged estimates can yield biased results [31], [32], so estimates of the absolute per-patient cost were derived by sampling from the posterior distribution of the regression coefficients and taking the mean of the exponent of these sampled values, with 95% confidence intervals calculated as the 2·5^th^ and 97·5^th^ percentiles of the exponentiated values.

A similar approach was used to calculate estimates of the annual per-patient cost for each patient type. For a given patient type, we set the patient type dummy variables to their appropriate value for that patient type, as well as setting the clinic visit frequency and CD4 count frequency variables to their subgroup-specific means, as both of these variables differ by patient type. All other variables were set to their global mean (mean across all observations), and the mean and confidence intervals for the annual per-patient cost calculated by simulating from the posterior distribution of the regression coefficients, as described above. To calculate total per-patient costs (including ARVs) we used current drug prices and regimen distributions for each country derived from the WHO Global Price Reporting Mechanism [33], with a 8.3% mark-up for transportation and other supply-chain management costs [12].

Exploratory analyses revealed that the size of the treatment program (as measured by patient volume) was strongly related to per-patient costs, with larger sites exhibiting substantially lower costs than smaller sites when controlling for other covariates. As a consequence, the per-patient cost calculated as an average across sites will be larger than the same statistic calculated as an average across patients. For an audience interested in budgeting and resource planning, it is intuitive that total funding requirements can be calculated by multiplying total patient volume by some measure of the average per-patient cost. For this purpose calculating the average cost across sites will give a biased (over)estimate of total costs, and the ‘patient-average’ cost alone is appropriate. For this reason, all dollar-valued results were calculated using this patient-average approach. This approach differs from prior analyses, which gave equal weight to each site when calculating summary statistics [12].

It was hypothesized that the effect of the distal determinants (location, health system level, type of administration) on per-patient costs would be mediated, in whole or in part, by their influence on the proximal determinants. For this reason three different model specifications were used to investigate the influence of the distal determinants. First, a parsimonious model was fit including only the distal determinants. A second model was then fit including these variables as well as variables relating to site maturity and patient volume. Finally, a full model was fit including the distal determinants as well as all proximal determinants. All regression models were implemented using the GLMM framework described above.

## Results

### Crude Site-Level Costs

The cost per patient varied widely in the sample, with annualized costs for adult established ART patients ranging from $36 to $4,374, with an interquartile range of $154–$586 and a median of $322.

### Proximal Determinants of Per-Patient Costs

Table 2 presents coefficients and measures of uncertainty for the regression of the logged per-patient cost against the proximal explanatory variables described in Table 1.

**Table 2:** GLMM regression of annual per-patient HIV treatment costs on proximal cost determinants.†

	Coefficient	Std. Error	95% CI	P-Value^‡^
Site Maturity	−0·055	0·011	(−0·076, −0·035)	<0·001***
Site Maturity Sq.	0·0008	0·0002	(0·0004, 0·0013)	<0·001***
Patient Volume	−0·166	0·057	(−0·277, −0·057)	0·004**
Patient Volume Sq.	0·006	0·003	(0·001, 0·011)	0·01*
Clinic Visit Frequency	0·037	0·006	(0·024, 0·048)	<0·001***
CD4 Count Frequency	0·186	0·019	(0·149, 0·224)	<0·001***
Comprehensiveness of Care	0·094	0·055	(−0·009, 0·211)	0·09
Clinician:Patient Ratio	0·010	0·002	(0·007, 0·014)	<0·001***
Doctor:Clinician Ratio	−0·551	0·489	(−1·548, 0·360)	0·26
Pct Mgmt-Admin	0·013	0·008	(−0·002, 0·029)	0·10
Log Per-Capita GDP	0·280	0·113	(0·047, 0·490)	0·02*
Intercept	3·439	0·845	(1·770, 5·037)	0·003**

† HIV treatment costs represent economic costs of site-level service delivery in 2010 US dollars, excluding ARVs and national/regional overhead costs. Regression model also included dummy variables for patient type (reference  =  established adult ART patients), coefficients not shown. ^‡^ '***'denotes p<0·001, '**'denote p<0·01, and '*'denotes p<0·05.

Most proximal determinants show a statistically significant relationship with per-patient treatment costs. Greater site maturity, higher patient volume, less frequent clinical and laboratory monitoring, less clinical staff per patient, and lower price levels (proxied by log per-capita GDP) are all independently associated with lower per-patient costs. The comprehensiveness of care and the fraction of resources devoted to site-level management and administration are both positively associated with per-patient costs, though this relationship is only marginally significant. The ratio of doctors to other clinical staff (relevant to task-shifting efforts) does not appear to be associated with per-patient costs, though given the small sample size it is possible that this analysis would not identify an effect of small magnitude, if present.

The magnitude of the regression coefficients are difficult to interpret directly and for this reason a series of comparisons is presented in Table 3 describing the impact of various program changes on per-patient costs.

**Table 3:** Change in annual per-patient cost for established adult ART patients associated with changes in individual cost determinants.†

	Absolute Change	Percentage Change	P-Value^‡^
Site matures from 0 to 12 months	−$157 (−$271, -$71)	−41% (−52%, −28%)	<0·001***
Site matures from 12 to 24 months	−$55 ($–92, -$25)	−25% (−35%, −15%)	<0·001***
Patient volume increases from 500 to 5,000 patients	−$166 (−$287, -$57)	−43% (−63%, −18%)	0·004**
Patient volume increases from 5,000 to 10,000 patients	−$58 (−$92, -$16)	−28% (−47% −6·3%)	0·02*
One additional clinic visit per year	$4·12 ($2·50, $6·12)	1·8% (1·3%, 2·4%)	<0·001***
One additional CD4 test per year	$22 ($15, $31)	9·7% (7·7%, 12%)	<0·001***
One additional service in care package	$22 (-$3·68, $52)	10% (−1·7%, 23%)	0·09
One additional clinician per 1,000 patients	$2·35 ($1·42, $3·55)	1·1% (0·7%, 1·4%)	<0·001***
10 percentage point increase in the fraction of siteresources going to mgmt and admin	$32 (-$5·46, $78)	14% (−2·5%, 33%)	0·10
Annual per-capita GDP rises from $500 to $1,500	$65 ($13, $122)	37% (6·1%, 73%)	0·02*
Annual per-capita GDP rises from $1,500 to $5,000	$104 ($15, $231)	41% (6·7%, 83%)	0·02*

† HIV treatment costs represent economic costs of site-level service delivery in 2010 US dollars, excluding ARVs and national/regional overhead costs. Changes in per-patient costs calculated from a regression of per-patient costs against proximal cost determinants. Each comparison shows the consequence of change in a single determinant, holding all other determinants at their mean values. ^‡^P-values represent two-sided test for difference from zero, '***'denotes p<0·001, '**'denotes p<0·.01, and '*'denotes p<0·05.

**Figure 1:**
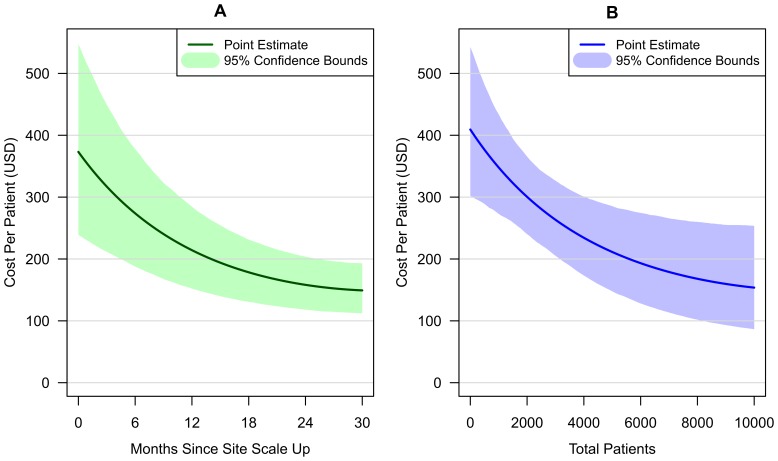
Change in annual per-patient cost for established adult ART patients as a function of site maturity and patient volume. Panel A shows annual HIV treatment cost as a function of site maturity. Panel B shows annual HIV treatment cost as a function of patient volume. HIV treatment costs represent annual economic costs of site-level service delivery in 2010 US dollars, excluding ARVs and national/regional overhead costs. Changes in per-patient costs calculated from a regression of per-patient costs against proximal cost determinants. Each panel shows the consequence of change in a single determinant, holding all other determinants at their mean values.

Patient volume and site maturity both have a substantial effect on per-patient costs, with an increase in each associated with considerably lower costs. A squared term was included in the regression equation for each of these variables. Both of these squared terms are positive, and the implications of this are presented in Figure 1, which shows the negative and convex relationship between per-patient costs and site maturity (first panel), and per-patient costs and patient volume (second panel), holding all other variables constant at their mean values.

**Figure 2:**
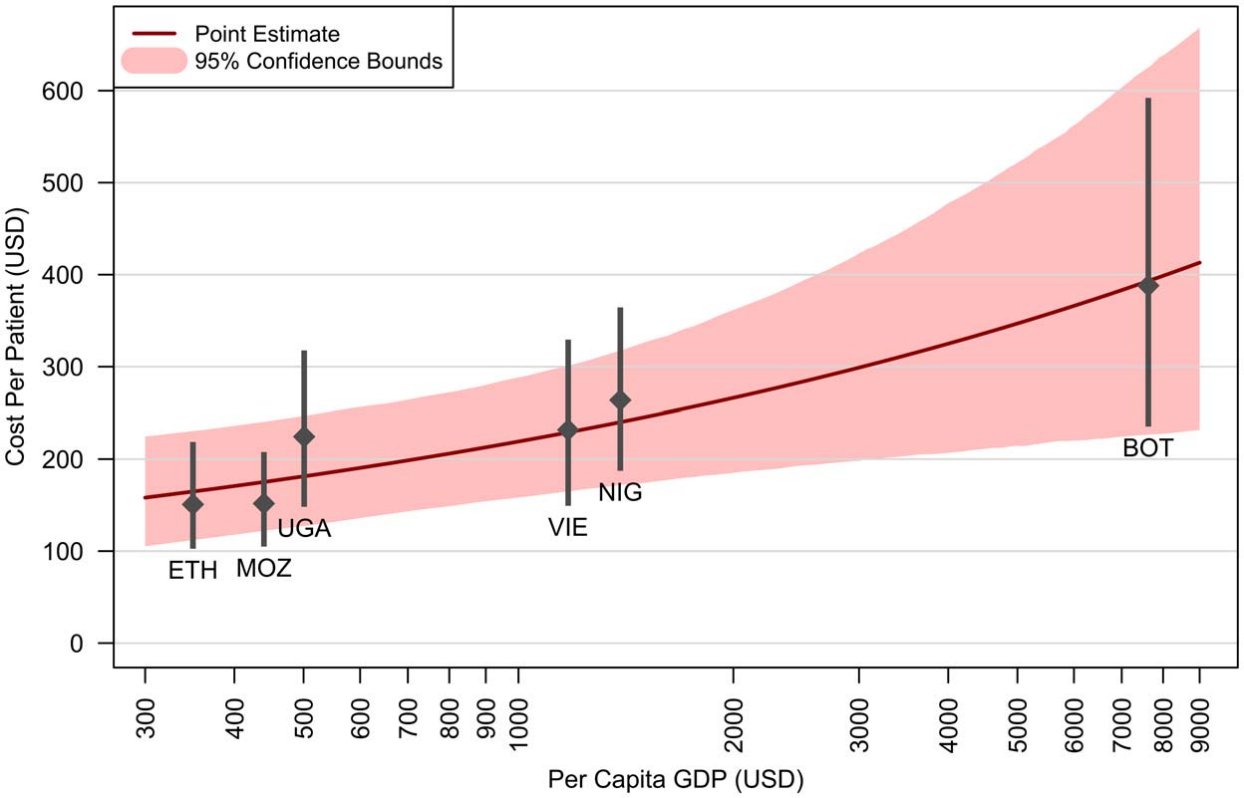
Change in annual per-patient cost for established adult ART patients as a function of per-capita GDP. HIV treatment costs represent economic costs of site-level service delivery in 2010 US dollars, excluding ARVs and national/regional overhead costs. Changes in per-patient costs calculated from a regression of per-patient costs against proximal cost determinants. The plot shows the consequence of changes in per-capita GDP, holding all other determinants at their mean values. Overplotted country-level estimates: ETH = Ethiopia, MOZ = Mozambique, UGA = Uganda, VIE = Vietnam, NIG = Nigeria, BOT = Botswana.

Per-patient costs are estimated to increase by approximately one-third for each one-unit increase in log per-capita GDP (equivalently, costs increase by 22% (4–41%) for every doubling of per-capita GDP). The implications of this in terms of absolute per-capita GDP are shown in Figure 2, which also shows the estimated mean and confidence interval for the per-patient cost in each country. These country-level results can be combined with current drug prices and regimen distributions for each country [33] to produce estimates of total per-patient costs for established adult ART patients on first-line regimens: $546 for Botswana, $261 for Ethiopia, $294 for Mozambique, $425 for Nigeria, $378 for Uganda, and $363 for Vietnam. The average across all countries was $365. The percentage of these totals attributable to non-ARV service delivery ranged from 51–71% across the 6 countries, with an overall average of 61%.

**Figure 3:**
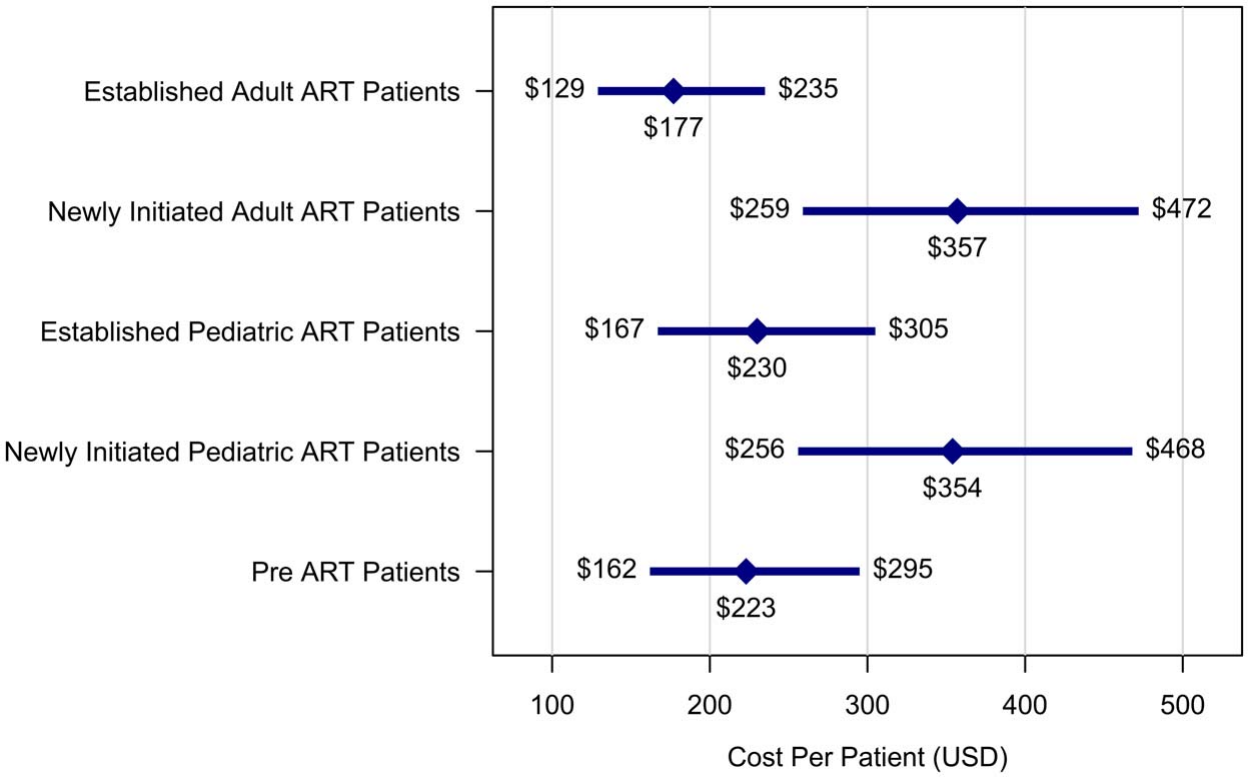
Average annual per-patient HIV treatment costs, by patient type. HIV treatment costs represent economic costs of site-level service delivery in 2010 US dollars, excluding ARVs and national/regional overhead costs. Cost estimates calculated from a regression of per-patient costs against proximal cost determinants. In figure, diamond signifies point estimate, length of bars signifies 95% confidence interval.

Figure 3 presents estimates of the per-patient cost for each patient type, averaged across all six countries. Newly initiated ART patients consume substantially more resources than established ART patients, and pre-ART patients substantially less. While the confidence intervals in the figure overlap, these differences are statistically significant, with established adult ART per-patients costs 26% (95% CI: 19–33%) higher than pre-ART per-patient costs, and newly initiated adult ART per-patient costs 58% (95% CI: 50%–67%) higher than established adult ART patients. Similarly, newly initiated pediatric ART per-patient costs are 56% (95% CI: 47%–64%) more costly than established pediatric ART patients, while the cost differences between adult and pediatric ART patients are small and non-significant.

### Distal Determinants of Per-Patient Costs

Three regression models were fit to investigate the impact of distal determinants (urban vs. rural location, health system level, and government vs. NGO/FBO) on per-patient costs: a parsimonious regression model just containing the three distal determinants, a second model containing these variables as well as site maturity and patient volume covariates, and a full model including all distal and proximal determinants. The results for these three regressions are shown in Table 4.

**Table 4:** GLMM regression of annual per-patient HIV treatment costs against distal cost determinants, with three model specifications.†

	Coefficient	Std. Error	95% CI	P-Value†
*Regression model includes distal determinants^‡^ plus patient type fixed effects (coefficients not shown)*				
Secondary level	0·17	0·32	(−0·47, 0·77)	0·60
Tertiary level	−0·20	0·31	(−0·78, 0·43)	0·50
Urban	−0·01	0·25	(−0·51, 0·45)	0·99
NGO/FBO	−0·24	0·34	(−0·92, 0·41)	0·51
*Regression model includes distal determinants, plus patient type fixed effects, site maturity and patient volume (coefficients not shown)*				
Secondary level	0·56	0·32	(−0·07, 1·18)	0·08
Tertiary level	0·38	0·33	(−0·24, 1·05)	0·24
Urban	−0·08	0·26	(−0·59, 0·44)	0·74
NGO/FBO	−0·14	0·34	(−0·83, 0·50)	0·67
*Regression model includes distal determinants, plus all proximal determinants included in earlier regressions (coefficients not shown)*				
Secondary level	0·18	0·29	(−0·42, 0·72)	0·52
Tertiary level	0·32	0·30	(−0·24, 0·93)	0·29
Urban	−0·11	0·24	(−0·60, 0·35)	0·61
NGO/FBO	−0·11	0·31	(−0·71, 0·52)	0·72

† HIV treatment costs represent economic costs of site-level service delivery in 2010 US dollars, excluding ARVs and national/regional overhead costs. P-values represent two-sided test for difference from zero, '***'denotes p<0·001, '**'denotes p<0·01, and '*'denotes p<0·05. ^‡^Distal determinants include health system level (primary vs. secondary vs. tertiary), location (urban vs. non-urban), and type of administration (government vs. NGO/FBO).

In none of these analyses do any of the distal determinants show a clear significant relationship with per-patient costs. While prior beliefs suggest that primary sites are more expensive than secondary and tertiary sites, such a relationship is not apparent in these data. The only set of results which approach statistical significance are in the regression controlling for site maturity and patient volume (middle panel in Table 4), where the estimates suggest that for a site of comparable size and maturity, primary sites might actually be cheaper. It could be that the economies of scale enjoyed by tertiary and secondary sites are (at least partially) offset by a more expensive care model, as secondary and tertiary sites grouped together are positively associated with the frequency of clinic visits (p = 0·31), frequency of CD4 tests (p = 0·05), number of different care services provided (p<0·001), and the clinician:patient ratio (p = 0·04), when controlling for all other cost determinants. While the small sample size means that non-significant findings must be interpreted with caution, it appears that these distal determinants–location, health system level, and type of administration–may have a weak relationship with per-patient costs.

## Discussion

The global funding devoted to HIV treatment dwarfs resources available for many other health concerns, but the goal of providing treatment access to all those in need has not yet been achieved. With constrained budgets, treatment programs must better understand the costs of care and identify opportunities to improve efficiency, in order to continue expanding treatment access. The results of these analyses reveal a number of possible paths to lower per-patient costs. The largest reductions in per-patient costs will likely be those that will be obtained with the least effort, as programs mature and treatment cohorts grow with the continued enrollment of patients. Given the strong relationship between program size and the per-patient cost, strategies that increase access through smaller sites may be more resource-intensive than a more centralized approach. While a focus on smaller sites may be important for achieving health sector goals of geographic equity and improved access in rural locations, these goals must be weighed against the additional cost. It is a notable finding of this analysis that, when controlling for patient volume and site maturity, primary-level sites appear to be no more expensive than sites at higher levels of the health system, and possibly less expensive as a result of a more-limited package of care. Other cost determinants are more amenable to policy intervention, such as extending the interval for clinical follow-up and laboratory monitoring of established patients, optimization of the care package accompanying ART, and standardizing staffing intensity. Each of these factors was found to make small but non-trivial contributions to total treatment costs.

When considering the policy applications of these findings, it is important to note that the outcome used in this analysis–the annual per-patient cost–does not capture the quality of care or extent of the health benefits enjoyed by patients receiving that care. As such, actions directed at reducing the per-patient cost must consider the possible impacts on quality and patient outcomes. The fact that universal access targets have not been reached except in a few settings provides strong motivation to treat as many patients as possible with the funding available, yet there will be a point at which the gains in terms of greater coverage will be more than offset by the harm to patient outcomes (if treatment quality falls) and program sustainability (if staff are overworked or infrastructure used beyond capacity).

The comprehensiveness of care index give an overall summary of how the number of additional care services contribute to total cost, yet does not distinguish between individual components of the care package, which may vary considerably in their costs and health impact. Other research has shown that some relatively inexpensive additions to the care package can produce health gains that more than justify their expense [34], [35]. For this reason the results of this analysis point to possible approaches for reducing costs but do not provide sufficient information to judge the cost-effectiveness of individual components of the care package. Additional research is needed to inform the question of how clinical outcomes might be affected by competing operational approaches or packages of care.

The results of this analysis provide an empirical basis for estimating the resource needs for supporting HIV treatment programs in the future, allowing analysts to begin to specify cost functions that are sensitive to program scale, maturity and other operating characteristics. While a second-best to empirical data collected within a given HIV treatment program, the relationship between per-capita GDP and per-patient costs identified in this analysis provides a method for creating approximate cost estimates for settings where a costing has not yet been undertaken or where the treatment program is still in its nascent stages. For such resource needs projections, it will be important to consider two cost categories excluded from this analysis: the cost of antiretroviral medications, and the costs of program administration incurred at regional and national-level. Each of these cost categories will represent a non-trivial fraction of the overall cost of a treatment program, and both will likely be governed by a set of determinants different from those assessed in this analysis. In the case of ARVs, the per-patient cost will be sensitive to drug prices and regimen distributions. As both of these factors can change rapidly, cost projections will need to be based on real-time data, and ideally account for changes in these factors that might be anticipated over the timeframe of the cost projections. Higher-level program administration costs will likely be subject to maturity and scale effects as well as the interactions of major program funders, but as yet little is known about these costs and they represent an important subject for future research.

Some limitations should be noted. The analysis does not include patient time-and-travel costs, which may be higher under a centralized scale-up approach. Also excluded from this analysis is the epidemic impact of infections averted through treatment, and the associated savings that would accrue from the averted infections. Similarly, productivity gains that result from improved health of patients, and averted HIV-associated orphanhood from reduction in AIDS deaths are also not included. Consideration of these broader societal costs and benefits might influence decisions about optimal treatment scale-up.

As programs gain more evidence about the cost and benefits of different clinical approaches, they will be better positioned to focus on high-impact services and streamline other aspects of care, freeing up resources to support larger patient cohorts. In this way the benefits of improved efficiency will be measured in the number of additional patients who can be enrolled on treatment, and through the resulting impact on HIV patients’ health, their families’ welfare, and the reduction in new HIV infections.
