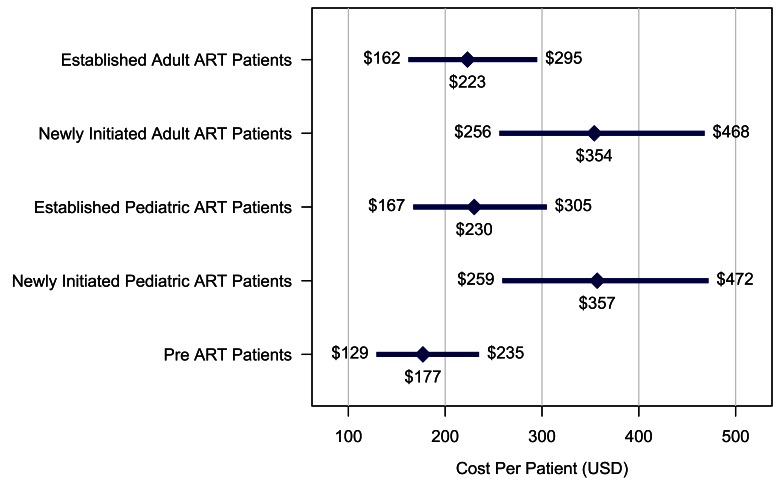# Correction: The Determinants of HIV Treatment Costs in Resource Limited Settings

**DOI:** 10.1371/annotation/1b6115d9-272e-4623-8ef7-265cd8e5aa28

**Published:** 2013-05-10

**Authors:** Nicolas A. Menzies, Andres A. Berruti, John M. Blandford

There was an error in Figure 3.

The correct version is available here: 

**Figure:**